# Antagonists of retinoic acid receptors (RARs) are potent growth inhibitors of prostate carcinoma cells

**DOI:** 10.1054/bjoc.2001.1939

**Published:** 2001-08

**Authors:** L A Hammond, C H Van Krinks, J Durham, S E Tomkins, R D Burnett, E L Jones, R A S Chandraratna, G Brown

**Affiliations:** Divisions of Cancer Studies and of, University of Birmingham Medical School, Edgbaston, Birmingham, B15 2TT, UK; Immunity and Infection, University of Birmingham Medical School, Edgbaston, Birmingham, B15 2TT, UK; Retinoid Research, Allergan Inc., Irvine, California, USA

**Keywords:** RAR antagonists, retinoic acid receptors, prostate cancer, growth inhibition, apoptosis

## Abstract

Novel synthetic antagonists of retinoic acid receptors (RARs) have been developed. To avoid interference by serum retinoids when testing these compounds, we established serum-free grown sub-lines (>3 years) of the prostate carcinoma lines LNCaP, PC3 and DU145. A high affinity pan-RAR antagonist (AGN194310, K_d_ for binding to RARs = 2–5 nM) inhibited colony formation (by 50%) by all three lines at 16–34 nM, and led to a transient accumulation of flask-cultured cells in G1 followed by apoptosis. AGN194310 is 12–22 fold more potent than *all-trans* retinoic acid (ATRA) against cell lines and also more potent in inhibiting the growth of primary prostate carcinoma cells. PC3 and DU145 cells do not express RARβ, and an antagonist with predominant activity at RARβ and RARγ (AGN194431) inhibited colony formation at concentrations (∼100 nM) commensurate with a K_d_ value of 70 nM at RARγ. An RARα antagonist (AGN194301) was less potent (IC_50_ ∼200 nM), but was more active than specific agonists of RARα and of βγ. A component(s) of serum and of LNCaP-conditioned medium diminishes the activity of antagonists: this factor is not the most likely candidates IGF-1 and EGF. In vitro studies of RAR antagonists together with data from RAR-null mice lead to the hypothesis that RARγ-regulated gene transcription is necessary for the survival and maintenance of prostate epithelium. The increased potencies of RAR antagonists, as compared with agonists, suggest that antagonists may be useful in the treatment of prostate carcinoma. © 2001 Cancer Research Campaign http://www.bjcancer.com


British Journal of Cancer (2001) 85(3), 453–462
© 2001 Cancer Research Campaign
doi: 10.1054/ bjoc.2001.1939, available online at http://www.idealibrary.com on IDEAL®

### Antagonists of retinoic acid receptors (RARs) are potent growth inhibitors of prostate carcinoma cells

**LA Hammond[1], CH Van Krinks[2], J Durham[2], SE Tomkins[2], RD Burnett[3], EL Jones[1], RAS Chandraratna[3] and G Brown[2]**

Divisions of [1]Cancer Studies and of [2]Immunity and Infection, University of Birmingham Medical School, Edgbaston, Birmingham B15 2TT, UK and [3]Retinoid Research, Allergan Inc., Irvine, California, USA

**Summary** Novel synthetic antagonists of retinoic acid receptors (RARs) have been developed. To avoid interference by serum retinoids when testing these compounds, we established serum-free grown sub-lines (>3 years) of the prostate carcinoma lines LNCaP, PC3 and DU145. A high affinity pan-RAR antagonist (AGN194310, $K_d$ for binding to RARs = 2–5 nM) inhibited colony formation (by 50%) by all three lines at 16–34 nM, and led to a transient accumulation of flask-cultured cells in G1 followed by apoptosis. AGN194310 is 12–22 fold more potent than *all-trans* retinoic acid (ATRA) against cell lines and also more potent in inhibiting the growth of primary prostate carcinoma cells. PC3 and DU145 cells do not express RARβ, and an antagonist with predominant activity at RARβ and RARγ (AGN194431) inhibited colony formation at concentrations (~100 nM) commensurate with a $K_d$ value of 70 nM at RARγ. An RARα antagonist (AGN194301) was less potent ($IC_{50}$ ~200 nM), but was more active than specific agonists of RARα and of βγ. A component(s) of serum and of LNCaP-conditioned medium diminishes the activity of antagonists: this factor is not the most likely candidates IGF-1 and EGF. In vitro studies of RAR antagonists together with data from RAR-null mice lead to the hypothesis that RARγ-regulated gene transcription is necessary for the survival and maintenance of prostate epithelium. The increased potencies of RAR antagonists, as compared with agonists, suggest that antagonists may be useful in the treatment of prostate carcinoma. © 2001 Cancer Research Campaign http://www.bjcancer.com

**Keywords**: RAR antagonists; retinoic acid receptors; prostate cancer; growth inhibition; apoptosis

Prostate cancer is one of the most common cancers in European and American males and has a high mortality rate (Landis et al, 1998; Rosenthal, 1998). Early disease is managed conservatively in Europe although in selected patients surgery is curative. In advanced disease, androgen ablation can produce symptomatic improvement, but the tumour often re-emerges in an androgen-independent and incurable form. One proposed treatment for androgen-independent prostate carcinoma is differentiation therapy, in which agents such as all-*trans*-retinoic acid (ATRA), 9-*cis*-retinoic acid and 1α, 25-dihydroxyvitamin $D_3$ ($D_3$) are used to induce growth arrest, differentiation and sometimes apoptosis of the tumour cells (Liang et al, 1999; de Vos et al, 1997; Skowronski et al, 1994; Hedlund et al, 1997).

ATRA and synthetic analogues exert their biological effects by binding to two families of retinoid receptors, the RARs (RARα, RARβ and RARγ) and the RXRs (RXRα, RXRβ and RXRγ). These are ligand-dependent transcription factors of the steroid/thyroid hormone nuclear receptor superfamily (Chambon 1994; Boehm et al, 1995). Activated RARs bind to retinoic acid-responsive elements (RAREs) in the promoters of genes and thus regulate gene expression. They also antagonize the activity of the transcription factor AP1 and thereby down-regulate the expression of some AP1-activated genes (Nagpal et al, 1995). One approach to unravelling the contributions of RAR sub-types to these actions has been the development of synthetic retinoids with selective activities at individual RARs and RXRs. For example, the diaryl-acetylenic retinoids allow separation of transactivation and AP1 antagonism functions at RARα (Nagpal et al, 1995). Recently, compounds that are selective for particular RAR sub-types have been synthesized: these include both agonists and antagonists (Chandraratna, 1995; Teng et al, 1999; Johnson et al, 1999). The functional selectivity of these compounds may open the way to achieving differentiation or apoptosis of tumour cells with minimal systemic side effects (Chandraratna, 1995).

Cell lines that are commonly used in the evaluation of investigational agents for prostate cancer include LNCaP, DU-145 and PC-3. LNCaP cells are androgen-responsive and express RARα, RARβ and RARγ, whereas DU-145 and PC3 cells are androgen-insensitive and do not express RARβ (Campbell et al, 1998). These lines are relatively insensitive to growth inhibition and induction of apoptosis by ATRA and by synthetic agonists. ATRA induces apoptosis of LNCaP cells at 2 μM (Gao et al, 1999), but neither inhibits the growth nor induces apoptosis of PC-3 and DU-145 cells at 2 μM (Lu et al, 1997 and 1999). Fenretinide (4HPR) induces apoptosis of LNCaP and of PC-3 cells at 1–5 μM and 25 μM, respectively (Pienta et al, 1993; Heish and Wu, 1997). Pfahl and colleagues examined the growth inhibitory effects of a panel of receptor-selective retinoids against DU145 and PC-3 cells and achieved complete growth inhibition and induction of apoptosis between 1 μM and 10 μM (Lu et al, 1999). They screened more than 100 retinoids for growth-inhibitory activity against prostate carcinoma lines: three RARγ agonists were the most active (Lu et al, 1999).

*Received 25 January 2001*
*Revised 3 May 2001*
*Accepted 8 May 2001*

*Correspondence to:* LA Hammond

**Table 1** Structures of novel retinoid analogues, and their receptor binding and transactivation properties

| | | | RARα | | RARβ | | RARγ | |
|---|---|---|---|---|---|---|---|---|
| Compound no. (AGN) | Structure | Receptor Specificity[1] | $K_d$[2] (nM) | $EC_{50}$[3] (nM) | $K_d$[2] (nM) | $EC_{50}$[3] (nM) | $K_d$[2] (nM) | $EC_{50}$[3] (nM) |
| 194078 |  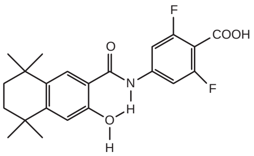 | RARα agonist | 4 | 140 | > 5000 | WA | > 5000 | NA[4] |
| 195153 |  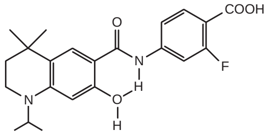 | RARα agonist | 40 | 130 | > 5000 | WA | > 5000 | WA[4] |
| 190299 |  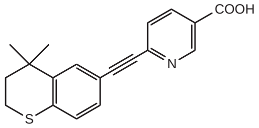 | RARβγ agonist | 616 | > 1000 | 41 | 18 | 57 | 42 |
| 194310 |  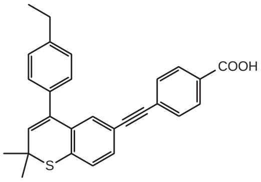 | RARαβγ antagonist | 3 | NA[4] | 2 | NA[4] | 5 | NA[4] |
| 194301 |  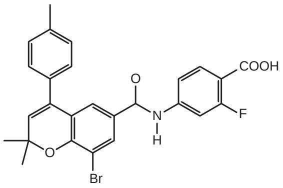 | RARα antagonist | 3 | NA[4] | 320 | NA[4] | 7250 | NA[4] |
| 194431 |  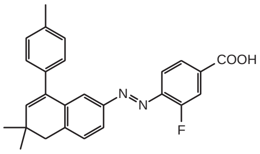 | RARβγ antagonist | 300 | NA[4] | 6 | NA[4] | 70 | NA[4] |

[1]When tested against RXRα, RXRβ and RXRγ, all compounds were inactive: $K_d$ values for binding were >10 μM, and they showed no activity in transactivation assays. [2]Binding to baculovirus-expressed RARα, RARβ and RARγ was measured. [3]$EC_{50}$ values for transactivation by RARs were determined by doubly transfecting CV-1 cells (500 cells/well) with an RAR reporter plasmid (ΔMTV-$TRE_p$-LUC) and one of the human RAR expression vectors, and then treating the transfected cells with the defined ligand (Nagpal et al, 1995). [4]Abbreviations: NA, inactive; WA, weak partial agonist.

In this study, we have investigated the extent to which synthetic retinoids directed against RAR sub-types inhibit the growth of prostate carcinoma cell lines. These compounds included agonists of RARα and RARβγ and antagonists of RARα, RARβγ and RARαβγ. In our screens we have used sub-lines of LNCaP, PC-3 and DU145 cells that had been grown long-term in serum-free conditions: the development and characteristics of these lines are described. The use of serum-free grown lines avoids the complication introduced by the small amounts of naturally occurring retinoids in serum. We find that RAR antagonists are substantially more potent than agonists in inducing growth arrest both of prostate carcinoma cell lines and of primary prostate carcinoma cultures. As such, they are potentially useful in prostate cancer therapy.

### MATERIALS AND METHODS

### Retinoids

The novel synthetic RAR ligands were synthesized at Allergan Inc. (Irvine, CA). Their structures are shown in Table 1. They comprise RARα agonists (AGN194078, AGN195153), a RARβγ agonist (AGN190299) (Nagpal et al, 1995), a specific RARα antagonist (AGN194301) (Teng et al, 1999), an RARβγ antagonist (AGN194431) and an RAR pan-antagonist (AGN194310) (Johnson et al, 1999). Our definition of the term RAR antagonist is a compound that binds to the RARs (see Table 1) and that does not activate gene transcription (see Table 1) but instead blocks the gene transcriptional activity induced by ATRA and other RAR

 *© 2001 Cancer Research Campaign*

agonists (Johnson et al, 1999). All the above compounds, the pan-RAR agonist TTNPB ((E)-4-[2-(5, 6, 7, 8-tetrahydro-5, 5, 8, 8-tetramethyl-2-naphthalenyl-1-propenyl]-benzoic acid) and ATRA were stored as 10 mM stock solutions in 50% ethanol/50% dimethysulphoxide at –20˚C. Control cultures contained the solvent alone.

### Establishment of serum free-grown stocks of prostate carcinoma cell lines

The prostate carcinoma cell lines LNCaP, PC-3 and DU-145 were from the American Type Culture Collection (ATCC, Rockville, MD) and maintained in RPMI 1640 medium (Gibco-BRL, Paisley, UK) supplemented with 10% fetal bovine serum (FBS, Gibco), 100 U/ml penicillin and 100 µg/ml streptomycin. Cells were grown at 37˚C in a humidified atmosphere of 5% $CO_2$ in air and passaged by trypsinizing with trypsin-EDTA (Gibco-BRL).

Serum grown LNCaP, PC-3 and DU-145 (FBS cells) were adapted to growth in serum-free conditions by replacing half of the medium at each medium change and passage with RPMI 1640 medium containing antibiotics (as above) and supplemented with insulin, transferrin, selenium dioxide, linoleic acid, and bovine serum albumin ($ITS^+$, Sigma, Poole, UK). All three lines readily adapted to growth under serum-free conditions ($ITS^+$ cells) without adverse effects on cell growth, viability and adherence. Serum and serum-free grown lines were sub-cultured in a similar manner. Following trypsinization of the serum-free grown lines, trypsin was inactivated using $ITS^+$ medium.

### Growth of primary prostate carcinoma cells

Core biopsies were obtained from patients undergoing investigation for suspected prostatic carcinoma. Consent for the use of this material for research purposes was obtained from the Local Research Ethics Committee and from the patients. Histological examination confirmed that the biopsies contained tumour material. Cultures were established essentially as described by Peehl and co-workers (Peehl et al, 1991), using the serum-free medium Prostate Epithelial Cell Growth Medium (PrEGM) supplemented with SingleQuots (Biowhittaker, Wokingham, UK). Cells were sub-cultured onto plates coated with collagen-1 (Greiner, Stonehouse, UK), and these plates and the above medium were used for assays.

### Analysis of the effects of retinoids on cell growth

In the clonal proliferation assay, single-cell suspensions of cells were plated at 400 (FBS cells) and 1200–1500 ($ITS^+$ cells) cells per 65 $mm^2$ dish in 4 ml of medium. The difference in the number of cells plated takes into account the lower cloning efficiency of the serum-free grown cells (see Results section). Retinoids ($10^{-9}$–$10^{-6}$ M) were added immediately and the medium plus retinoids were replaced at day 2. Plates were incubated (see above) for 14 days (serum-free grown cells) or 9 days (serum-grown cells), fixed using 1% formaldehyde in 0.9% NaCl, stained with 1% methylene blue in 0.9% saline and allowed to dry. Around 200 colonies were counted on each plate. Colony size was variable and the minimum number of cells in a cluster regarded as a colony was 70. When agents had blocked colony formation, even very small clusters of cells were not visible on the plates. Plates were set up in duplicate, and at least three experiments of each type were performed. Colony formation on agent-treated plates is presented as a percentage (mean ± SE) of the number of colonies on untreated plates. Vehicle alone had no effect on colony formation (see Results).

To further characterize the effect of the pan-RAR antagonist AGN194310 on LNCaP $ITS^+$ cells, bulk cultures were seeded at $2–5 \times 10^5$ cells per 75 $cm^2$ flask. AGN194310 was added immediately and at day 2. Adherent cells were harvested by trypsinization, pooled with those in suspension, and counted.

### Measurements of cell cycle status and of apoptosis

Cells, harvested by trypsinization, were stained with propidium iodide and the distribution of cells within phases of the cell cycle was determined using a Becton-Dickinson Flow Cytometer and CellFIT Cell-Cycle Analysis software. Apoptotic cells were identified, within pooled adherent and suspension cells, by the TUNEL assay (Gorczyca et al, 1993). A FITC-conjugated antibody to bromodeoxyuridine (Becton-Dickinson & Co., Mountain View, CA) was used to identify cells labelled with bromodeoxyuridine triphosphate and green fluorescence was measured by FACS analysis at 510–550 nm.

### Immunodetection of RARs in $ITS^+$-grown cells

Cell pellets were resuspended in lysis buffer (2% (w/v) SDS, 60 mM Tris-HCl (pH 6.6), 5 mM EDTA, 10 mM DTT, 1 mM PMSF) and boiled for 10 min. Samples (90 µg) were electrophoresed on 12% SDS-PAGE gels, electroblotted to Hybond-ECL membranes (Amersham Life Sciences, Bucks, UK) and probed using polyclonal antibodies to RARα, RARβ, and RARγ, diluted 1/1000 (kindly provided by P. Chambon (RARα) and Santa Cruz Biotechnology Inc. Wembley, Middlesex, UK (RARβ and RARγ)). Blots were visualized by enhanced chemiluminescence using an HRP-conjugated secondary antibody (Amersham Life Sciences).

### Statistical analysis

The effects of individual agents on colony formation were compared to control untreated and vehicle treated cultures using the Student's *t*-test.

### RESULTS

### Binding and transactivation properties of retinoids

Table 1 summarizes RAR binding and transactivation by the retinoids investigated for activity against prostate carcinoma cells. None of the compounds bound to baculovirus-expressed RXRα, RXRβ and RXRγ ($K_d$ > 10 K) or transactivated an RXR-responsive promotor construct (human CRBP II promotor DR1 elements) in the presence of RXR expression vectors (data not shown). Selectivity of analogues at RAR receptor subtypes was shown by in vitro binding experiments, using baculovirus-expressed RARα, RARβ and RARγ. Similar selectivity was observed in the RARα, RARβ and RARγ transactivation assays.

The RAR antagonists are of particular interest in this study (see below). AGN194310 is a pan-antagonist that binds to RARα, RARβ and RARγ with equal and high affinities ($K_d$ = 2–5 nM). AGN194301 is a high affinity RARα antagonist ($K_d$ = 3 nM); AGN194431 is RARβγ-selective, and binds more strongly to RARβ ($K_d$ = 6 nM) and RARγ ($K_d$ = 70 nM) than to

© 2001 Cancer Research Campaign 

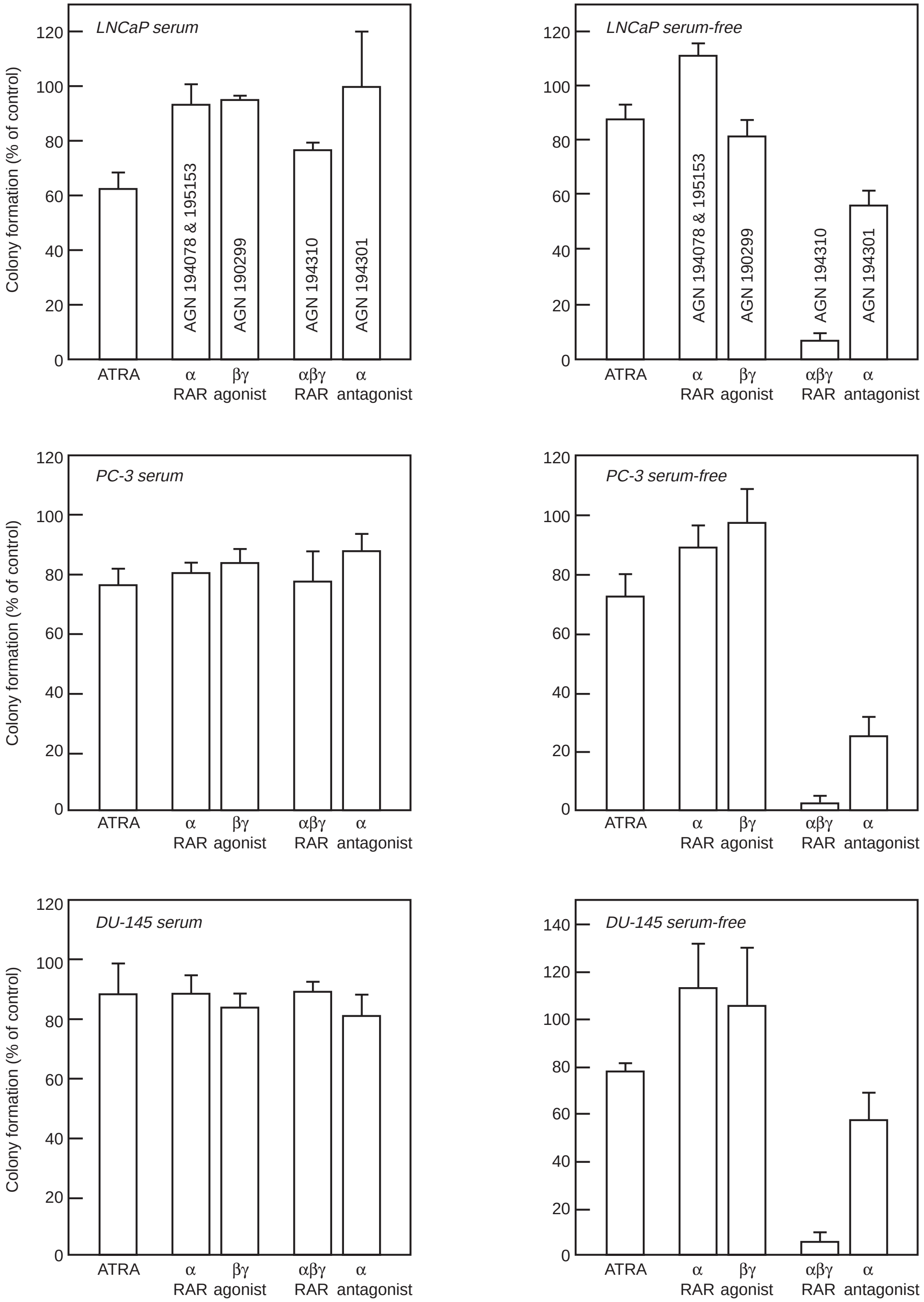

**Figure 1** RAR antagonists block colony formation by serum free-grown prostate carcinoma lines, but are less effective against serum-grown cells. Compounds were screened, all at 100 nM, for their ability to inhibit plate colony formation by serum free (ITS$^{+}$)- and serum (FBS)-grown LNCaP, DU145 and PC3 cells. Data obtained for compounds of identical receptor specificity were similar and have been combined. The compounds tested were: ATRA; RARα agonists, AGN194078 & AGN195153; RARβγ agonists AGN190168 & AGN190299; pan-RAR antagonist AGN194310 and RARα antagonist AGN194301. Data (means ± SE) are presented as a percentage of the number of colonies on untreated plates

RARα ($K_d$ = 300 nM). To confirm the activity of RAR antagonists in a model system, we investigated their effects on ATRA-induced differentiation of HL60 cells to neutrophils (Breitman et al, 1980). HL60 cells express RARα, and treatment with 100 nM ATRA for 5 days gave rise to 64 ± 5% neutrophils that displayed stimulated nitroblue tetrazolium reduction. AGN194310 (a pan-RAR antagonist) and AGN194301 (a RARα antagonist), added at 100 nM, prevented ATRA-induced neutrophil differentiation: the percentages of mature cells were 5% (AGN194310) and 1% (AGN194301).

 *© 2001 Cancer Research Campaign*

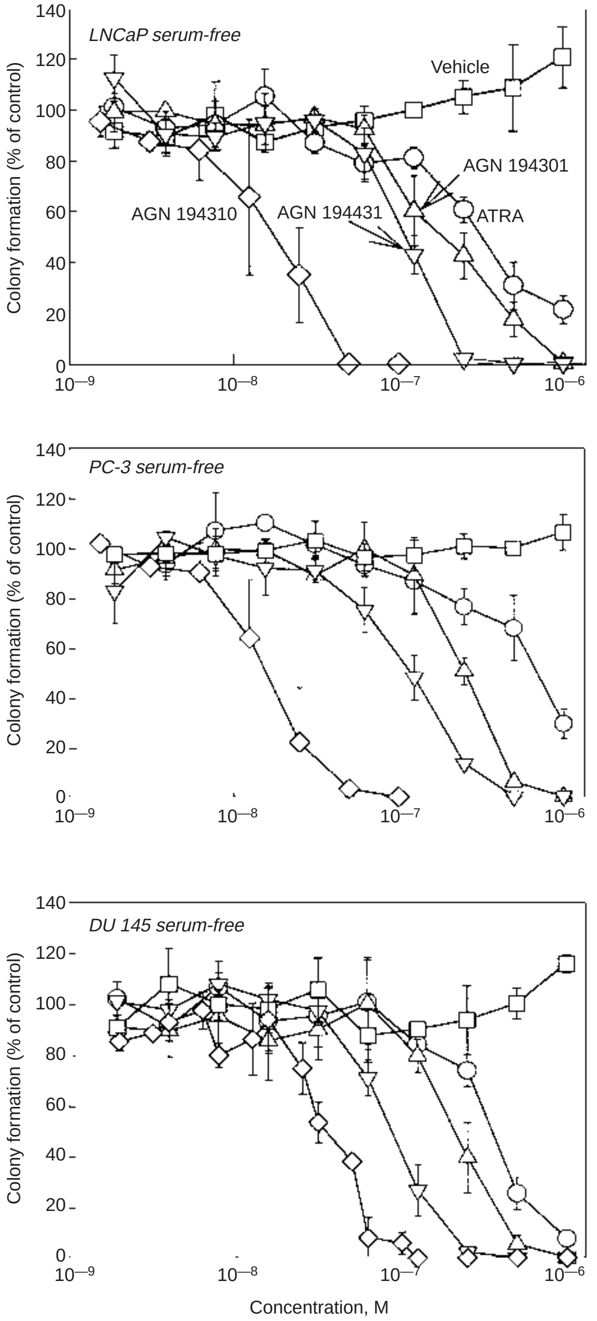

**Figure 2** A pan-RAR antagonist is a potent inhibitor of colony formation by serum-free grown prostate carcinoma cells. A pan-RAR antagonist AGN194310 (diamonds), an antagonist of RARβγ AGN194431 (inverted triangles), an antagonist of RARα AGN194301 (triangles), ATRA (circles) and appropriate vehicle controls (squares) were titrated against ITS+-grown LNCaP, DU145 and PC3 cells using the plate clonogenic assay. The effects of retinoids and vehicles are presented as a percentage of the number of colonies observed on untreated plates. Data are the means ± SE of values from at least three separate replicate experiments

### Characterization of ITS+-grown LNCaP, PC3 and DU145 prostate carcinoma lines

To prevent naturally occurring retinoids and other ligands from masking the effects of added retinoids, we developed serum-free stocks of the prostate carcinoma lines. These ITS+ lines have been maintained serum-free for ~3 years and are identical in appearance to their counterparts grown in FBS. ITS+ and FBS stocks grow

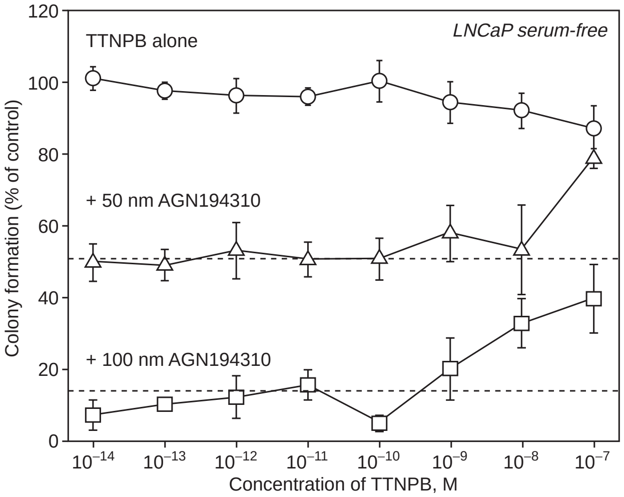

**Figure 3** A pan-RAR agonist counteracts the growth inhibitory effect of the pan-RAR antagonist. The pan-RAR agonist TTNPB was titrated, against LNCaP ITS+-grown cells using the plate clonogenic assay, alone (circles) and in combination with 50 nM (triangles) and 100 nM (squares) of the pan-RAR antagonist AGN194310. The hatched lines show the inhibitory effects of the two doses of AGN194310 alone. Data are presented as a percentage of the number of colonies observed on untreated plates and are the means ± SE of values from at least three separate replicate experiments with similar doubling times: 24 ± 4 h (ITS+) vs 24 ± 1 h (FBS) for LNCaP cells; 21 ± 1 h (ITS+) vs 29 ± 2 h (FBS) for DU145 cells; 17 ± 1 h (ITS+) vs 20 ± 1 h (FBS) for PC3 cells. Each ITS+ line has a lower cloning efficiency in the plate colony assay than its FBS counterpart. The percentage cloning efficiencies are: LNCaP cells, 13 ± 1 (ITS+) vs 19 ± 2.9 (FBS); DU145 cells, 10 ± 1 (ITS+) vs 15 ± 2 (FBS); and PC3 cells, 7 ± 1 (ITS+) vs 11 ± 2 (FBS).

The ITS+ cells expressed the same RAR receptors, determined by Western analysis, as the parental FBS cells. LNCaP cells expressed RARα, RARβ and RARγ and DU145 and PC3 cells expressed RARα and RARγ (data not shown, and Campbell et al, 1998). FBS-grown prostate carcinoma cell lines are reported to be relatively insensitive to growth inhibition by ATRA (de Vos et al, 1997; Lu et al., 1997; Gao et al., 1999; Lu et al., 1999), and we confirmed this for ITS+-grown and FBS-grown cells. A 50% inhibition of colony formation by the ITS+ cells was attained at 344 ± 32 nM ATRA for LNCaP, 402 ± 74 nM ATRA for DU145 and 419 ± 120 nM ATRA for PC3. Only modest inhibition of colony formation was observed with FBS-grown cells at 1 μM ATRA; reductions in colony formation by LNCaP, DU145 and PC3 cells were 49 ± 6%, <1% and 33 ± 7%, respectively.

### RAR antagonists inhibit colony formation by ITS+-grown prostate carcinoma lines

When compounds were screened, all at 100 nM, for their ability to inhibit colony formation, the most striking inhibition occurred when ITS+-grown cells were treated with the pan-RAR antagonist AGN194310 (Figure 1). Colony formation was reduced to 3–7% of control values in all three cell lines. AGN194310 inhibited colony formation by FBS-grown cells by only 11–23%. The RARα-specific antagonist AGN194301 also inhibited colony formation by the three prostate lines when grown in ITS+ medium (by 42–74%), but had little effect on FBS-grown cells (0–19% inhibition). Treatment of the three cell lines (ITS+ and FBS cells) with ATRA, RARα agonists and a RARβγ agonist had little effect

© 2001 Cancer Research Campaign on colony formation (Figure 1). LNCaP ITS+ and DU-145 ITS+ cells showed a very slight increase (~17% and 13%, respectively) in colony formation post-treatment with the RARα agonists. However, statistical analysis of the actual number of colonies seen on control (untreated) vs agonist-treated plates revealed that an increase in colony formation only approached significant in the case of LNCaP ITS+ cells ($P$ = 0.15) and was not significant in the case of DU-145 ITS+ cells ($P$ = 0.8).

To exclude the possibility that the inhibitory effects of the antagonists are merely cytotoxic effects on all targets, we tested whether the pan-RAR antagonist AGN194310 and the RARα-specific antagonist AGN194301 interfere with the clonal growth of the promyeloid cell line HL60 (Wallington et al, 1996). Serum-free grown HL60 cells (>7 years) were plated as single cells in microtitre wells and treated with 100 nM of the above agents. The number of viable cells was enumerated daily for 4 days. AGN194310 and AGN194301 did not adversely affect the clonal growth of HL60 cells; the cloning efficiencies of untreated and antagonist-treated cultures were the same (90–93%, and see Wallington et al, 1996). Additionally, the numbers of viable cells, at day 4, in control ($n$ = 78), AGN194310-treated ($n$ = 65) and AGN194301-treated ($n$ = 67) cultures were 18, 17 and 22, respectively. Viabilities of the single cells in all three conditions were >95%.

We therefore focused our attention on inhibition of colony formation by ITS+-grown LNCaP, PC3 and DU-145 cells by RAR antagonists. Figure 2 shows dose-response curves for the pan-RAR antagonist (AGN194310) and for antagonists of RARβγ (AGN194431) and of RARα (AGN194301). Once again, AGN194310 potently inhibited colony formation by all three lines, with $IC_{50}$ values of 16 ± 5 nM for LNCaP cells; 18 ± 6 nM for PC3 cells; and 34 ± 7 nM for DU-145 cells. The RARβγ antagonist AGN194431 was less potent than AGN194310: $IC_{50}$ values were 99 ± 10 nM for LNCaP cells, 104 ± 2 nM for PC3 cells and 88 ± 12 nM for DU145 cells. The RARα antagonist AGN194301 was the least potent; $IC_{50}$ values were 203 ± 36 nM (LNCaP), 235 ± 20 nM (PC3) and 201 ± 23 nM (DU145).

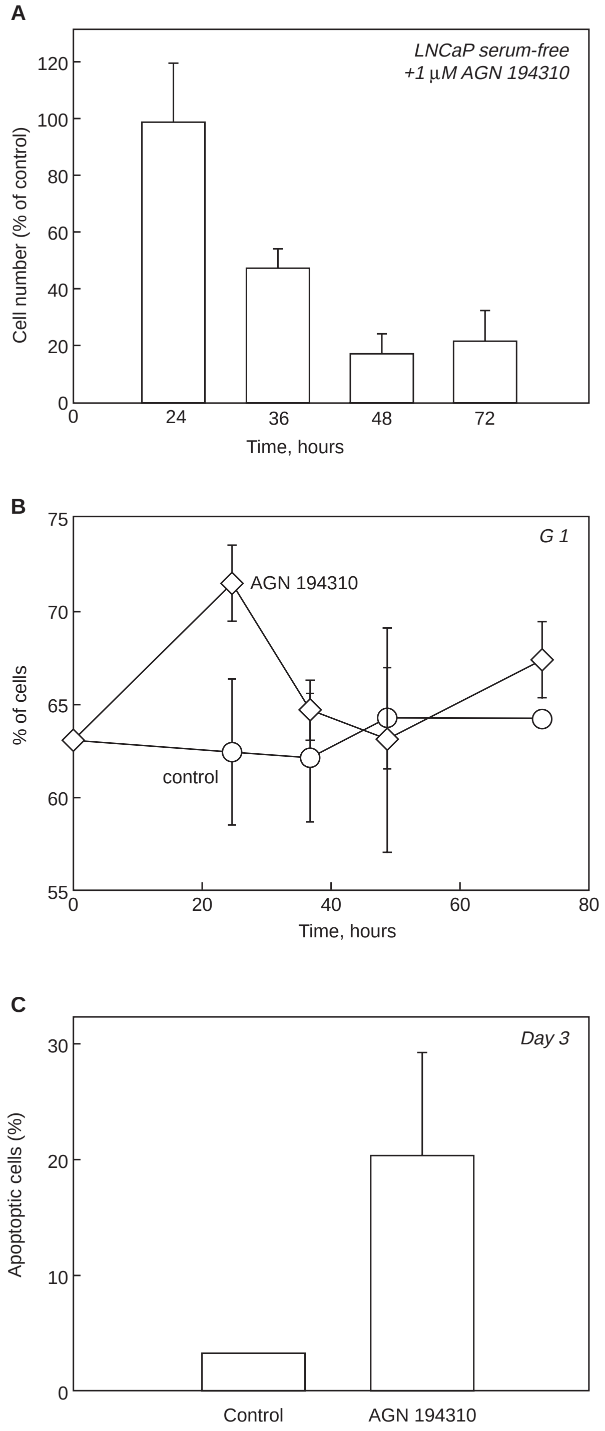

**Figure 4** Pan-antagonism of RARs leads to a rise in the proportion of G1 cells followed by apoptosis. Data obtained for the effect of 1 µM of the pan-antagonist AGN194310 on viable cell numbers are presented as a percentage of the cells present in untreated flask cultures of ITS+-grown LNCaP cells (**4A**). The proportions of pan-antagonist-treated (diamonds) and untreated (circles) cells in each phase of the cell cycle were determined by propidium iodide staining and flow cytometry (**4B**). Apoptotic cells were identified by TdT end-labelling and flow cytometry (**4C**). Data are the means ± SE of values from triplicate experiments

### The pan-RAR agonist TTNPB counteracts the growth inhibitory effect of the pan-RAR antagonist AGN 194310

In these experiments, we used the non-metabolized RAR agonist TTNPB to avoid any influence of down-stream metabolites. TTNPB, when used alone at concentrations up to $10^{-7}$ M, had only a small inhibitory effect on the clonal growth of LNCaP ITS+ cells (~10% inhibition at $10^{-7}$ M, see Figure 3). A higher concentration of TTNPB ($10^{-6}$ M) inhibited colony formation (by >90%). The pan-RAR antagonist AGN194310 was tested for its ability to inhibit colony formation at concentrations of 50 nM and 100 nM alone and in combination with $10^{-7}$ to $10^{-14}$ M TTNPB. As shown in Figure 3, 50 nM and 100 nM AGN194310 inhibited colony formation to 41% and 14% of control values, respectively. When this agent was used together with 100 nM TTNPB, there was almost complete reversal of the growth inhibitory effect of 50 nM AGN194310 (see Figure 3) and partial reversal of the effect of 100 nM AGN194310 (colony formation increased from 14% to 40% of control values). This reversal, together with the finding that RAR antagonists block agonist-induced differentiation of HL60 cells (see above), show that antagonists are binding to and mediating their effects via RARs.

### RAR antagonism causes cells to accumulate in G1 and undergo apoptosis

Eighty percent (±11%) growth inhibition of cells grown in flasks for 3 days was achieved by treatment with 1 µM AGN194310

 © 2001 Cancer Research Campaign

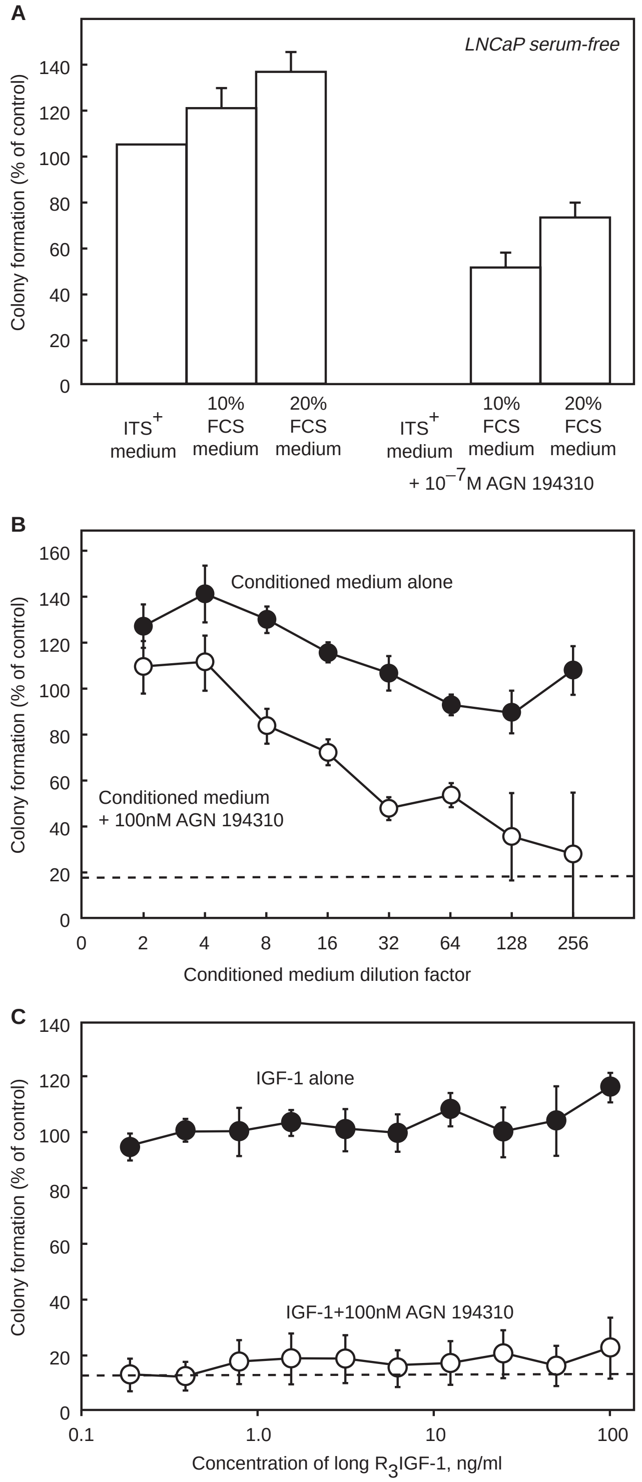

**Figure 5** A serum component that is also produced by LNCaP ITS+ cells blocks pan-antagonist-mediated inhibition of colony formation. (**A**) shows that plating LNCaP ITS+ cells in 10% and 20% FBS stimulates colony formation and that the inhibitory effect of 100 nM of the pan-RAR antagonist AGN194310 is partially blocked. (**B**) shows that conditioned medium from exponentially growing cultures of LNCaP ITS+ cells stimulates colony formation to a small degree (open circles) and completely blocks the inhibitory effect of 100 nM AGN194310 (closed circles). (**C**) shows that IGF-1 neither stimulates colony formation by LNCaP ITS+ cells (closed circles) nor interferes with the inhibitory effect of 100 nM AGN194310 (closed circles). The hatched lines in (**B**) and (**C**) show the effect of AGN194310 alone. Data are the means ± SE of values from triplicate experiments

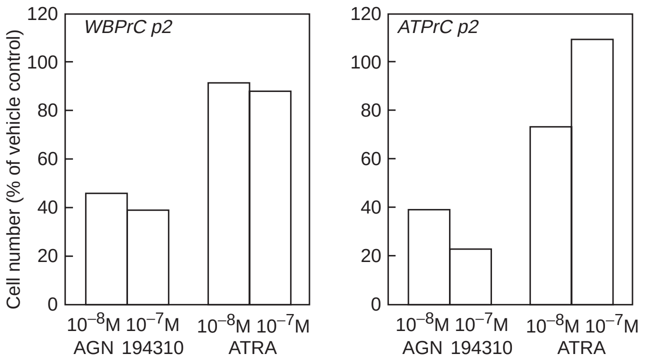

**Figure 6** The pan-RAR antagonist is more potent that ATRA in inhibiting growth of primary prostate carcinoma cells. Bulk cultures of carcinoma cells from core biopsies of two patients with prostatic carcinoma were treated on day 0 with either 10 and 100 nM of the pan-RAR antagonist AGN194310, 10 and 100 nM ATRA or vehicles for these two agents. On days 3 (ATPrC) and 6 (WBPrC), adherent cells were harvested, pooled with any suspension cells and viable cells were counted. Agent effects are presented as a percentage of the number of viable cells in untreated cultures. Data are from single experiments with duplicate cultures and the means of values are shown.

(Figure 4A). Analysis of cell cycle status by propidium iodide labelling revealed a transient increase, at day 1, in the proportion of G1 cells from a control value of 62 ± 4% to 71 ± 2% for AGN194310-treated cultures (Figure 4B). The proportion of cells in S phase decreased from 22 ± 2% (control cultures) to 15 ± 3% (AGN194310-treated). By day 3, a substantial proportion of the pan-antagonist-treated cells had detached from the flask and were undergoing apoptosis (20 ± 9% vs. 3 ± 1% in control cultures), as shown by TdT end-labelling and FACS analysis (Figure 4C).

### A component(s) of serum and of LNCaP-conditioned medium blocks the growth-inhibitory effect of RAR antagonists on ITS+-grown cells

Our results indicated that FBS-grown and ITS+-grown cells were either intrinsically different in their sensitivities to RAR antagonists or a serum component blocked the action of these agents. To investigate the latter possibility, we measured inhibition of colony formation when ITS+-grown LNCaP cells were plated in FBS medium. Plating ITS+-grown cells in 10% and 20% FBS medium modestly stimulated colony formation (by 15 ± 8% in 10% FBS and by 30 ± 8% in 20% FBS). The addition of serum to ITS+-grown LNCaP cells partially reversed the inhibition of colony formation by AGN194310. Colony formation was < 1% when ITS+ cells were plated in ITS+ medium and treated with 100 nM AGN194310, and was restored to 48 ± 6% and 69 ± 6% by the presence of 10% FBS and 20% FBS, respectively (Figure 5A). Similarly, treatment of LNCaP ITS+-grown cells in ITS+ medium with the RARα antagonist AGN194301 (100 nM) inhibited colony formation by 69 ± 16% and colony formation was restored to 55 ± 16% and 119 ± 5% of control values respectively, by addition of 10% or 20% FBS.

When analysing growth inhibition by AGN194310, it became apparent that a higher concentration (1 μM) was required to attain maximum growth inhibition of the flask cultures than of the fewer cells plated in the colony assay (100 nM). To investigate whether LNCaP cells might produce something that interferes with the action of the antagonist, we determined whether conditioned medium from exponentially growing LNCaP ITS+ cultures could block the growth inhibitory effect of AGN194310. Figure 5B

**Table 2** Comparison of the growth inhibitory activities ($IC_{50}$) and of the receptor affinities ($K_d$) of antagonists

| Cell line | Receptors expressed | AGN194310 $\alpha \sim \beta \sim \gamma$ $K_d\ \alpha = 3$ nM $K_d\ \beta = 2$ nM $K_d\ \gamma = 5$ nM | AGN194431 $\beta > \gamma >> \alpha$ $K_d\ \alpha = 300$ nM $K_d\ \beta = 6$ nM $K_d\ \gamma = 70$ nM | AGN4301 $\alpha >> \beta/\gamma$ $K_d\ \alpha = 3$ nM $K_d\ \beta = 320$ nM $K_d\ \gamma = 7250$ nM |
|---|---|---|---|---|
| LNCaP | RAR α,β,γ | $IC_{50}$ = 16 nM | $IC_{50}$ = 99 nM | $IC_{50}$ = 203 nM |
| PC-3 | RARα,γ | $IC_{50}$ = 18 nM | $IC_{50}$ = 104 nM | $IC_{50}$ = 235 nM |
| DU145 | RAR α,γ | $IC_{50}$ = 34 nM | $IC_{50}$ = 88 nM | $IC_{50}$ = 201 nM |

shows that plating the LNCaP ITS[+] cells in LNCaP-conditioned medium abrogated the growth inhibitory activity of AGN194310. This effect was concentration-dependent, with half-maximal reversal of the inhibition at a 1:10 dilution of the conditioned medium (Figure 5B).

Insulin-like growth factor-1 (IGF-1) is one serum component that has been reported by Pietrzkowski and collegues to be produced by LNCaP, PC-3 and DU145 cells grown in serum-free medium. These workers have proposed that these cells grow by an autocrine loop in which overproduced IGF-1 activates its receptor (Pietrzkowski et al, 1993). Other investigators have identified elevated plasma IGF-1 as a predictor of prostate cancer risk (Chan et al, 1998; Manztzoros et al, 1997). In our hands, the long R3 form of IGF-1 (0.1–100 ng/ml) (Gropep, Adelaide, Australia), which does not bind to IGF binding proteins, had no effect on colony formation by LNCaP ITS[+] cells, with or without 100 nM AGN194310 (see Figure 5C). The IGF-1 used was biologically active: it stimulated growth of the breast carcinoma line MCF-7 in 10% charcoal-stripped serum by 64 ± 27% (Xie et al, 1997). Long R3-IGF-1 also slightly stimulated the growth of FBS-grown LNCaP cells in charcoal-stripped serum (by 23 ± 5%).

Epidermal growth factor (EGF)-mediated signalling is also thought to be a key autocrine regulator of growth in prostate cancer and EGF stimulates the growth of LNCaP and DU-145 cells under serum-free conditions (Sherwood et al, 1998). EGF (at 1, 10 and 20 ng/ml) (R & D, Abingdon, UK) did not reverse the inhibition of colony formation by 100 nM AGN194310 (>95% inhibition), but did stimulate the growth of FBS-grown LNCaP cells in charcoal-stripped serum (by 25 ± 5%).

### AGN194310 inhibits the growth of primary prostate carcinoma cells

We established primary cultures of carcinoma cells from core biopsies of two patients with prostatic carcinoma. Cultures had epithelial morphology, confirmed by immunocytochemical staining for cytokeratins and prostate-specific antigen, and contained few contaminating fibroblasts. Flask cultures of these cells were tested, at passage 2, for their sensitivity to AGN194310 and to ATRA. Cell growth in vehicle-treated and ATRA-treated cultures was similar to controls. However, treatment of both primary lines with AGN194310 (10 and 100 nM) caused substantial growth inhibition (see Figure 6).

### DISCUSSION

RAR antagonists potently inhibit the growth of three prostate carcinoma cell lines; the most potent was a pan-RAR antagonist (AGN194310), followed by an antagonist with predominant activity at RARβγ (AGN194431) and the RARα antagonist (AGN194301). The pan-RAR antagonist AGN194310 was 12–22-fold more potent than ATRA. However, growth inhibition at low antagonist concentrations was only observed using cells that were grown in the absence of serum. Fanjul and co-workers have shown that RAR antagonists (three compounds) inhibit the growth of breast carcinoma lines more potently than ATRA, but only in low serum (0.5%) conditions (Fanjul et al, 1998). Their most potent compound was MX781, a pan-RAR antagonist, which abolished cell proliferation at >1 μM. AGN194310 appears to be more potent than MX781 against carcinoma cells since it largely prevents colony formation at 100 nM. In our studies, and those of Fanjul and co-workers, the growth inhibitory activities of ATRA and of agonists selective for RARα and for RARβγ were similar when cells were grown serum-free or in 0.5% FBS vs 10% FBS.

At which RAR subtype(s) does antagonism compromise the growth and survival of prostate carcinoma cells? Table 2 summarizes $IC_{50}$ and $K_d$ values for antagonists and the RAR types expressed by the cell lines. PC-3 and DU-145 cells do not express detectable RARβ, so the RARβγ antagonist probably inhibits the growth of these cells through RARγ and the $IC_{50}$ values observed (75–104 nM) are close to the Kd for binding to isolated RARγ (~70 nM). The pan-RAR antagonist also inhibits growth at concentrations ($IC_{50}$ 16–34 nM) close to its $K_d$ for binding to RARγ (5 nM). These results suggest that antagonism of RARγ is sufficient to compromise survival and growth, at least of PC-3 and DU145 cells. The RARα-selective antagonist AGN194301 only inhibited colony formation at concentrations (~200 nM) much higher than its $K_d$ for binding to RARα (3 nM), and it has far too low an affinity for RARγ for this to be its target.

Information on the influence of RARs in controlling the growth of prostate cells has come from studies of the RAR-null mice (Lohnes et al, 1993; Lufkin et al, 1993). Mature RARγ-null mice show atrophy and squamous metaplasia of the prostate and seminal vesicles (Lohnes et al, 1993), whereas the RARα-null mouse shows no such defects (Lufkin et al, 1993). The prostates of normal mice contain only RARγ, and RARα and RARβ did not appear in the RARγ-null prostates. These results demonstrate the need for RAR function in the prostate, which is fulfilled by RARγ in the mouse. Taken together, the mouse studies and our observations suggest that RARγ-regulated gene transcription is necessary for the survival and maintenance of normal prostate epithelium in both mouse and human. Our studies using serum-free grown cells suggest that a basal or very low level of RARγ-mediated transcription is important to cell survival. Like other RARγ-directed antagonists and inverse agonists, AGN194310 probably represses basal levels of receptor-mediated transcription by increasing association between RARγ and the nuclear co-repressor N-CoR (Klein et al, 2000).

Treatment of LNCaP cells with the pan-RAR antagonist AGN194310 led to apoptosis, as does treatment with the RAR agonists ATRA (Gao et al, 1999) and fenretinide (Pienta et al, 1993). That both agonists and antagonists may induce apoptosis via RARs suggests that a particular level of RAR-mediated transcriptional activity may be essential for cell survival; and that disturbing this balance in either direction may result in cell death. Alternatively, RAR antagonists are active at concentrations close to their $K_d$ values, whereas growth inhibition by agonists requires amounts that are >100× greater than their $K_d$s, suggesting that some of the effects of agonist treatment may not reflect selective actions at RARs. Indeed, Piedrafita and Pfahl concluded that induction of apoptosis in Jurkat cells (a T cell line) by the RARγ

 © 2001 Cancer Research Campaign agonist CD437 is not transcriptionally mediated since levels of apoptosis are unaffected by inhibitors of protein and of mRNA synthesis (Piedrafita and Pfahl, 1997). Moreover, ATRA binds to and enhances activity of the transmembrane mannose-6-phosphate/IGF-2 receptor (Kang et al, 1998a and 1998b): this might suppress cell growth via activation of TGF-β.

RAR antagonists were more effective than ATRA in inhibiting the growth of all three prostate carcinoma lines grown serum-free. However, an unidentified component of serum partially reverses the activity of RAR antagonists. Moreover, serum-free grown LNCaP cells produce a factor(s) that interferes with the activity of RAR antagonists, and which also stimulates colony formation by LNCaP cells. Thus, the inhibitory effect of RAR antagonism on cell growth may be over-ridden by an autocrine growth-stimulatory loop. IGF-1 and EGF did not reverse the effect of RAR antagonism, so future investigations should aim to identify the 'autocrine factor(s)', which may limit the therapeutic effectiveness of RAR antagonists.

Despite this possible problem, the potency of the pan-RAR antagonist AGN194310 against primary carcinoma cells indicates that antagonists may be particularly useful therapeutic agents. Human primary prostate carcinoma cells express RARα and RARγ, and occasionally RARβ (Lotan et al, 2000). Moreover, Gyftopoulos and co-workers have shown that RARα expression correlates to some extent with tumour grade and that expression is more profound in highly proliferative tumours (Gyftopoulos et al, 2000). As to selecting a retinoid for possible clinical use, a pan-RAR antagonist, such as AGN194310, may be the most effective growth inhibitory agent if each of the RARs can functionally maintain human prostate carcinoma cells and RARα expression is important to proliferative status. Further information as to the effectiveness of the AGN194310 compound against a range of patient primary material and in vivo mouse model systems of prostate carcinoma is important to the development of this compound.

### ACKNOWLEDGEMENTS

This work was supported by a grant from Allergan Inc. Irvine, CA. We are grateful to Mr DMA Wallace for the provision of primary material and to Bob Michell for his valuable comments.

### REFERENCES

Boehm MF, Heyman RA, Patel S, Stein RB and Nagpal S (1995) Retinoids: biological function and use in the treatment of dermatological diseases. *Exp Opin Invest Drugs* **4**: 593–612

Breitman TR, Selonick SE and Collins SJ (1980) Induction of differentiation of the human promyelocytic leukaemia cell line (HL-60) by retinoic acid. *Proc Natl Acad Sci USA* **77**: 2936–2940

Campbell MJ, Park S, Uskokovic MR, Dawson MI and Koeffler HP (1998) Expression of retinoic acid receptor-β sensitises prostate cancer cells to growth inhibition mediated by combinations of retinoids and 19-*nor* hexafluoride vitamin $D_3$. *Endocrinology* **139**: 1972–1979

Chambon P (1994) The retinoid signalling pathways: molecular and genetic analyses. *Semin Cell Biol* **5**: 115–125

Chan JM, Stampfer MJ, Giovannucci E, Gann PH, Ma J, Wilkinson P, Hennekens CH and Pollack M (1998) Plasma insulin-like growth factor-1 and prostate cancer risk: a prospective study. *Science* **279**: 563–566

Chandraratna RAS (1995) Tazarotene – first of a new generation of receptor-selective retinoids. *Br J Dermatol* **135**: 18–25

de Vos S, Dawson MI, Holden S Le T, Wang A, Cho SK, Chen DL and Koeffler HP (1997) Effects of retinoid X receptor-selective ligands on proliferation of prostate cancer cells. *Prostate* **32**: 115–121

Fanjul AN, Piedrafita FJ, Al-Shamma H and Pfahl M (1998) Apoptosis induction and potent antiestrogen receptor-negative breast cancer activity *in vivo* by a retinoid antagonist. *Cancer Res* **58**: 4607–4610

Gao M, Ossowski L and Ferrari AC (1999) Activation of Rb and decline in androgen receptor protein precede retinoic acid induced apoptosis in androgen-dependent LNCaP cells and their androgen-independent derivative. *J Cell Physiol* **179**: 336–346

Gorczyca W, Bigman K, Mittleman A, Ahmed T, Gong J, Melamed MR and Darzynkiewicz Z (1993) Induction of DNA strand breaks associated with apoptosis during treatment of leukaemias. *Leukaemia* **7**: 659–670

Gyftopoulos K, Perimenis P, Sotiropoulou-Bonikou G, Sakellaropoulos G, Varakis I and Barbalias GA (2000) Immunohistochemical detection of retinoic acid receptor-alpha in prostate carcinoma: correlation with proliferative activity and tumour grade. *Int Urol Nephrol* **32**: 263–269

Hedlund TE, Moffatt KA, Uskokovic MR and Miller GJ (1997) Three synthetic vitamin D analogues induce prostate-specific acid phosphatase and prostate specific antigen while inhibiting the growth of human prostate cancer cells in a vitamin D receptor- dependent fashion. *Clin Cancer Res* **3**: 1331–1338

Heish TC and Wu JM (1997) Effects of fenretinide (4-HPR) on prostate LNCaP cell growth, apoptosis and prostate-specific gene expression. *Prostate* **33**: 97–104

Johnson AT, Wang L, Standeven AM, Escobar M and Chandraratna RAS (1999) Synthesis and biological activity of high-affinity retinoic acid receptor antagonists. *Bioorganic and Medicinal Chemistry* **7**: 1321–1338

Kang JX, Bell J, Leaf A, Beard RL and Chandraratna RAS (1998a) Retinoic acid alters the intracellular trafficking of the mannose-6-phosphate/insulin-like growth factor-II receptor and lysosomal enzymes. *Proc Natl Acad Sci USA* **95**: 13687–13691

Kang JX, Li Y and Leaf A (1998b) Mannose-6-phosphate/insulin-like growth factor-II receptor is a receptor for retinoic acid. *Proc Natl Acad Sci USA* **95**: 13671–13676

Klein ES, Wang JW, Khalifa B, Gavigan SA and Chandraratna RAS (2000) Recruitment of nuclear corepressor and coactivator to the retinoic acid receptor by retinoid ligands. *J Biol Chem* **275**: 19401–19408

Landis SH, Murray T, Bolden S and Wingo PA (1998) Cancer statistics. *Cancer J for Clinicians* **48**: 6–29

Liang JY, Fontana JA, Rao JN, Ordonez JV, Dawson MI, Shroot B, Wilber JF and Feng P (1999) Synthetic retinoid CD437 induces S-phase arrest and apoptosis in human prostate cancer cells LNCaP and PC-3. *Prostate* **38**: 228–236

Lohnes D, Kastner P, Dierich A, Mark M, LeMeur M and Chambon P (1993) Function of retinoic acid receptor γ in the mouse. *Cell* **73**: 643–658

Lotan Y, Xu XC, Shalev M, Lotan R, Williams R, Wheeler TM, Thompson TC and Kadmon D (2000) Differential expression of nuclear retinoid receptors in normal and malignant prostates. *J Clin Oncol* **18**: 116–121

Lu X-P, Fanjul A, Picard N, Pfahl M, Rungta D, Nared-Hood K, Carter B, Piedrafita FJ, Tang S, Fabbrizio E and Pfahl M (1997) Novel retinoid-related molecules as apoptosis inducers and effective inhibitors of human lung cancer cells in vivo. *Nature Med* **6**: 686–690

Lu X-P, Fanjul A, Picard N, Shroot B and Pfahl M (1999) A selective retinoid with high activity against an androgen-resistant prostate cancer cell type. *Int J Cancer* **80**: 272–278

Lufkin T, Lohnes D, Mark M, Dierich A, Gorry P, Gaub MP, LeMeur M and Chambon P (1993) High postnatal lethality and testis degeneration in retinoic acid alpha mutant mice. *Proc Natl Acad Sci USA* **90**: 7225–7229

Manztzoros CS, Tzonou A, Signorello LB, Stampfer M, Trichopoulos D and Adami HO (1997) Insulin-like growth factor 1 in relation to prostate cancer and benign prostatic hyperplasia. *Br J Cancer* **76**: 1115–1118

Nagpal S, Athankar J and Chandraratna RAS (1995) Separation of transactivation and AP1 antagonism functions of retinoic acid receptor α. *J Biol Chem* **270**: 923–927

Peehl DM, Wong ST, Terris MK and Stamey TA (1991) Culture of prostatic epithelial cells from ultrasound-guided needle biopsies. *Prostate* **19**: 141–147

Piedrafita FJ and Pfahl M (1997) Retinoid-induced apoptosis and Sp1 cleavage occur independently of transcription and require caspase activation. *Mol Cell Biol* **17**: 6348–6358

Pienta JK, Nguyen NM and Lehr JE (1993) Treatment of prostate cancer in the rat with the synthetic retinoid fenretinide. *Cancer Res* **53**: 224–226

Pietrzkowski Z, Mulholland G, Gomella L, Jameson BA, Wernicke D and Baserga R (1993) Inhibition of the growth of prostatic cancer cell lines by peptide analogues of insulin-like growth factor 1. *Cancer Res* **53**: 1102–1106

Rosenthal DS (1998) Changing trends (editorial). *Cancer J for Clinicians* **48**: 3–4

Sherwood ER, Van Dongen JL, Wood CG, Liao S, Kozlowski JM and Lee C (1998) Epidermal growth factor receptor activation in androgen-independent but not androgen-stimulated growth of human prostatic carcinoma cells. *Br J Cancer* **77**: 855–861

© 2001 Cancer Research Campaign 

Skowronski RJ, Peehl DM and Feldman D (1994) Vitamin D and prostate cancer: 1,25-dihydroxyvitamin $D_3$ receptors and actions in human prostate cancer cell lines. *Cancer Res* **54**: 805–810
Teng M, Duong TT, Johnson AT, Klein ES, Wang L, Khalifa B and Chandraratna RAS (1999) Identification of highly potent retinoic acid receptor α-selective antagonists. *J Medicinal Chemistry* **40**: 2445–2451
Wallington LA, Durham J, Bunce CM and Brown G (1996) Growth of single HL60 cells in liquid culture: Analysis of the influences of differentiative agents. *Leukemia Research* **20**: 821–829
Xie SP, James SY and Colston KW (1997) Vitamin D derivatives inhibit the mitogenic effects of IGF-1 on MCF-7 human breast cancer cells. *J Endocrinol* **154**: 495–504

© 2001 Cancer Research Campaign
